# Multimodal mucosal and systemic immune characterization of a non-human primate trachoma model highlights the critical role of local immunity during acute phase disease

**DOI:** 10.1371/journal.pntd.0012388

**Published:** 2024-08-02

**Authors:** Elodie Paulet, Vanessa Contreras, Mathilde Galhaut, Ida Rosenkrands, Martin Holland, Matthew Burton, Jes Dietrich, Anne-Sophie Gallouet, Nathalie Bosquet, Francis Relouzat, Sébastien Langlois, Frank Follmann, Roger Le Grand, Marc Labetoulle, Antoine Rousseau

**Affiliations:** 1 Université Paris-Saclay, Inserm, CEA, Center for Immunology of Viral, Auto-immune, Hematological and Bacterial diseases (IMVA-HB/IDMIT), Fontenay-aux-Roses & Le Kremlin-Bicêtre, France; 2 Center for Vaccine Research, Statens Serum Institut, Copenhagen, Denmark; 3 Clinical Research Department, London School of Hygiene and Tropical Medicine, London, United Kingdom; 4 International Centre for Eye Health, London School of Hygiene and Tropical Medicine, London, United Kingdom; 5 Service d’Ophtalmologie, Hôpital Bicêtre, Assistance Publique-Hôpitaux de Paris, Le Kremlin Bicêtre, France; 6 Service d’Ophtalmologie, Hôpital National de la Vision des 15-20, IHU Foresight, Paris, France; Medical University of Vienna, AUSTRIA

## Abstract

**Background:**

Trachoma is a leading cause of infection-related blindness worldwide. This disease is caused by recurrent *Chlamydia trachomatis* (Ct) infections of the conjunctiva and develops in two phases: i) active (acute trachoma, characterized by follicular conjunctivitis), then long-term: ii) scarring (chronic trachoma, characterized by conjunctival fibrosis, corneal opacification and eyelid malposition). Scarring trachoma is driven by the number and severity of reinfections. The immune system plays a pivotal role in trachoma including exacerbation of the disease. Hence the immune system may also be key to developing a trachoma vaccine. Therefore, we characterized clinical and local immune response kinetics in a non-human primate model of acute conjunctival Ct infection and disease.

**Methodology/Principal findings:**

The conjunctiva of non-human primate (NHP, Cynomolgus monkeys—*Macaca fascicularis-*) were inoculated with Ct (B/Tunis-864 strain, B serovar). Clinical ocular monitoring was performed using a standardized photographic grading system, and local immune responses were assessed using multi-parameter flow cytometry of conjunctival cells, tear fluid cytokines, immunoglobulins, and Ct quantification. Clinical findings were similar to those observed during acute trachoma in humans, with the development of typical follicular conjunctivitis from the 4^th^ week post-exposure to the 11^th^ week. Immunologic analysis indicated an early phase influx of T cells in the conjunctiva and elevated interleukins 4, 8, and 5, followed by a late phase monocytic influx accompanied with a decrease in other immune cells, and tear fluid cytokines returning to initial levels.

**Conclusion/Significance:**

Our NHP model accurately reproduces the clinical signs of acute trachoma, allowing for an accurate assessment of the local immune responses in infected eyes. A progressive immune response occurred for weeks after exposure to Ct, which subsided into a persistent innate immune response. An understanding of these local responses is the first step towards using the model to assess new vaccine and therapeutic strategies for disease prevention.

## Author summary

*Chlamydia trachomatis* is the leading infectious cause of blindness worldwide. The pathogenesis of trachoma is more complicated than other types of bacterial conjunctivitis: clinical signs of trachoma are rooted in repeated *Chlamydia trachomatis* infections of the inner eyelid surfaces, which roughens the skin. This leads to eyelid deformation causing the lashes to rub against the cornea. Over multiple years, this persistent rubbing leads to abrasion and eventually corneal opacification that can cause vision loss. The immune system is a pivotal aspect of this disease and is involved in disease aggravation and is also key for exploring the development of a trachoma vaccine. Here we describe a non-human primate model of trachoma that accurately reproduces acute human eye disease, allowing for a precise assessment of the local immune responses in infected eyes. We found that there was an ongoing immune response that continued for 4 weeks after exposure to *Chlamydia trachomatis*, which then subsided into a persistent innate immune response. Understanding these local responses is the first step towards using the model to assess new vaccines and therapeutic strategies to prevent disease.

## Introduction

Trachoma is currently the leading infectious cause of blindness worldwide. In 2022, trachoma was endemic in 42 countries (mainly in Africa), with 125 million people at risk, and 1.9 million visually-impaired [1,2]. The infection is caused by *Chlamydia trachomatis* (Ct), a Gram-negative obligate intracellular bacteria. The natural history of disease is divided into two successive phases: i) an acute (or active) phase characterized by follicular conjunctivitis, and ii) scarring (or chronic phase) characterized by conjunctival fibrosis, eyelid malposition, and trichiasis that ultimately causes corneal opacification [3,4]. Scarring or chronic trachoma develops over multiple years and after recurrent Ct infections [5].

The *World Health Organization* (WHO)’s SAFE (*Surgery*, *Antibiotic*, *Facial cleanliness*, *and Environmental changes*) strategy which is implemented by local health ministries in endemic zones, effectively decreased the prevalence of trachoma [6]. However, there are major drawbacks limiting the long-term efficacy of mass drug administration (MDA) preventive campaigns to combat trachoma. These include: i) off target antimicrobial resistance [7], ii) possible skewing of the gut microbiome [8], iii) multiple rounds of treatment are required in some populations [5,9], iv) a long term commitment from donor organizations to the programs, v) a need for (and sometimes lack of) continued surveillance and, vi) a lack of improvement in environmental and socio-economic conditions alongside MDA campaigns [10]. Taken together, the goal of eradication (rather than elimination) can only be achieved with a vaccine. Therefore a vaccine development strategy seems necessary to address the aforementioned drawbacks in endemic areas [9,11]. Previous vaccine development strategies with differing preclinical models have undergone human trials [12–15]. However, these trials have reported mixed outcomes [12–15]. A thorough understanding of the pathogenesis of trachoma and host-pathogen interactions are crucial for identifying correlates of protection and developing preventative strategies. Although the extrinsic etiological agent for trachoma is conjunctival Ct infection, immune-mediated inflammation is likely a substantial factor in pathogenesis [3,4,16–18]. The crosstalk between Ct infection and the immune response in the pathogenesis of trachoma, is complex and undeciphered. Recently, there have been significant advances in understanding the immune response during trachoma [16]. These include the essential role of non-specific and specific T cell responses for Ct clearance that causes concurrent tissue damage [18]. Other important insights include: studies of model infections that indicate neutrophil-mediated hyperinflammation has an adverse effect on ocular tissue damage and destruction [17,19] including, changes in disease biomarkers, such as local cytokines during the different stages of trachoma [20]. However, the majority of studies are cross-sectional, and published longitudinal data remains scarce. Hence a greater understanding of the pathogenesis of trachoma is required in order to evaluate potential Ct vaccines. Elucidating the precise mechanisms by which Ct triggers the clinical and biological changes in trachoma requires longitudinal data in a relevant animal model. In this regard, NHP models are especially interesting due to similarities with human immune responses and clinical manifestations [21,22]. We hypothesized that longitudinal follow-up with multimodal mucosal and systemic immune assessment would provide new insights into the pathogenesis of trachoma.

After a pilot study to validate the infectious dose needed to obtain clinical signs of trachoma, we optimized a NHP trachoma model that involved Ct ocular infection in macaques, which elicited follicular conjunctivitis that reproduced human features of acute trachoma [3,23,24]. We then characterized the dynamics of local immune responses over time using a combination of multiparameter flow cytometry performed on conjunctival cells, tear and serum cytokine analysis, and Ct-specific immunoglobulin quantification.

## Methods

### Ethics statement

Experimental protocols were approved by the institutional ethics committee (Comité d’éthique en experimentation animale—CetEA—#17_072 (Group 1) and #21_008 (Group 2)), and were authorized by the "Research, Innovation, and Education Ministry" under registration number APAFIS#720–201505281237660 (Group 1), and APAFIS#31037-2021041418019968v1 (Group 2). All experimental procedures adhered to the European guidelines for animal care (“Journal Officiel de l’Union Européenne”, directive 2010/63/UE, September 22, 2010), and according to CEA institutional guidelines.

### Animals

The study sample was comprised of 6 male (Group 1) and 12 female (Group 2) Cynomolgus monkeys (*Macaca fascicularis*). The age ranged from 57 to 97 months for Group 1, and 36 months to 38 months for Group 2. All animals were derived from AAALAC-certified primate breeding facilities in Mauritius, and were housed in IDMIT’s animal facility (CEA, Fontenay-aux-roses, France, authorization #D92-032-02, Préfecture des Hauts de Seine, France) in individual cages during the acute infectious phase, under BSL-2 containment.

Before inclusion into the study, animals had to have a negative test for salmonella, yersinia, shigella, herpes b, hepatitis b, SIV, STLV, tuberculosis, measles, flu and rabies. General observations were conducted daily to assess animal welfare that included evaluation of visual contact, active and interactive behavior, correct food and water intake, appearance of hair, and the absence of stereotypy. Additionally, body weight and rectal temperature were monitored at each sampling time-point (taking advantage of general anesthesia), along with a complete blood cell count (blood sampled with 1 mL EDTA tubes and analyzed with the DxH800 Beckman Coulter hematology analyzer for all standard blood cell count parameters). Sampling procedures were conducted under general anesthesia, performed using intramuscular tiletamine and zolazepam (Zoletil 100, 5mg/kg, Virbac, France). All sampling procedures were performed at the same time once a week or every second week, based upon the type of sample being collected.

### Bacterial strain, exposure and treatment

B/Tunis-864 strain (B serovar) of *Chlamydia trachomatis* (Ct) was provided by the Statens Serum Institut (SSI, Copenhagen, Denmark). Bacteria were titrated and stored at -80°C at a concentration of 8x10^5^ infection forming units (IFU)/μL, then diluted in SPG buffer (sucrose 0.2M, sodium phosphate 20mM, and glutamic acid 5mM, pH 7.4) before administration at a final concentration of either 2x10^5^ IFU/μL or 2x10^4^ IFU/μL for different groups. Animals were inoculated with 20 μL of diluted bacteria using a micropipette, in both the inferior and superior conjunctival fornices, bilaterally. Mock inoculated controls received only SPG buffer. At 7 weeks post-exposure (p.e.), Group 2 received oral azithromycin 20 mg/kg/day the first day then, 10 mg/kg daily for the next 2 days, according to the standard recommendations for the treatment of chlamydia infections. The azithromycin tablets were crushed and diluted in sterile water and administered to the animals via gavage [25] (Azithromycin 250 mg Sandoz, Sandoz, France). Oral administration was performed following mild sedation using 0.5 mg/kg intramuscular ketamine and 0.5mg/kg medetomidine (Ketamine, Virbac France, and Domitor, Orion, Finland) which was subsequently reversed with 0.25 mg/kg intramuscular atipamezole hydrochloride (Antisedan, Orion, Finland).

### Study design

**Fig 1:**
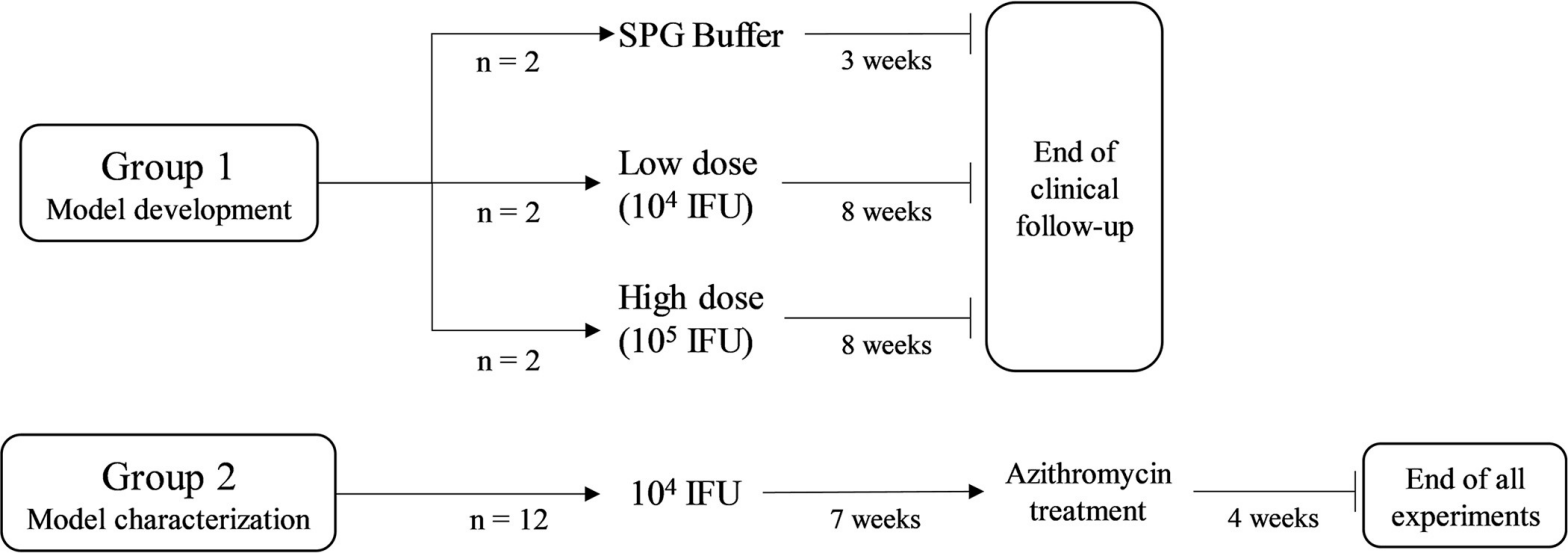
Study design. Group 1 was used to select an exposure dose while group 2 was used to characterize clinical manifestation, local and systemic immune response, and bacterial load. SPG stands for sucrose phosphate glutamic and IFU for infection forming units.

In a pilot study to define an appropriate challenge dose, and to determine a clinical grading scale, group 1 was exposed to 10^4^ (low dose, n = 2 macaques) or 10^5^ (high dose, n = 2) IFU/eye, or SPG buffer alone (SPG Buffer, n = 2 macaques), and evaluated with a standardized clinical follow-up throughout the study period,. The exposure dose of 10^4^ IFU/eye was then selected for further characterization of immune responses, clinical symptoms, and bacterial load profiles in group 2 (n = 12 macaques) (**Fig 1**). Both groups underwent identical standardized clinical follow-up throughout the study period.

In accordance with the replacement, reduction and refinement principles for animal research (3R guidelines), all macaques in group 2 were treated with azithromycin based on 2 considerations: prioritizing the welfare of the animal subjects and optimizing resource utilization. Specifically, these macaques were concurrently engaged in a complementary study necessitating antibiotic intervention to ensure effective bacterial eradication.

As the outcome of the study was classified as minimally harmful, the macaques were not euthanized as a result of this study. The end of experiments was defined as the end of clinical follow-up (i.e. conjunctival scoring) and sampling.

### Clinical scoring of the conjunctiva

**Fig 2:**
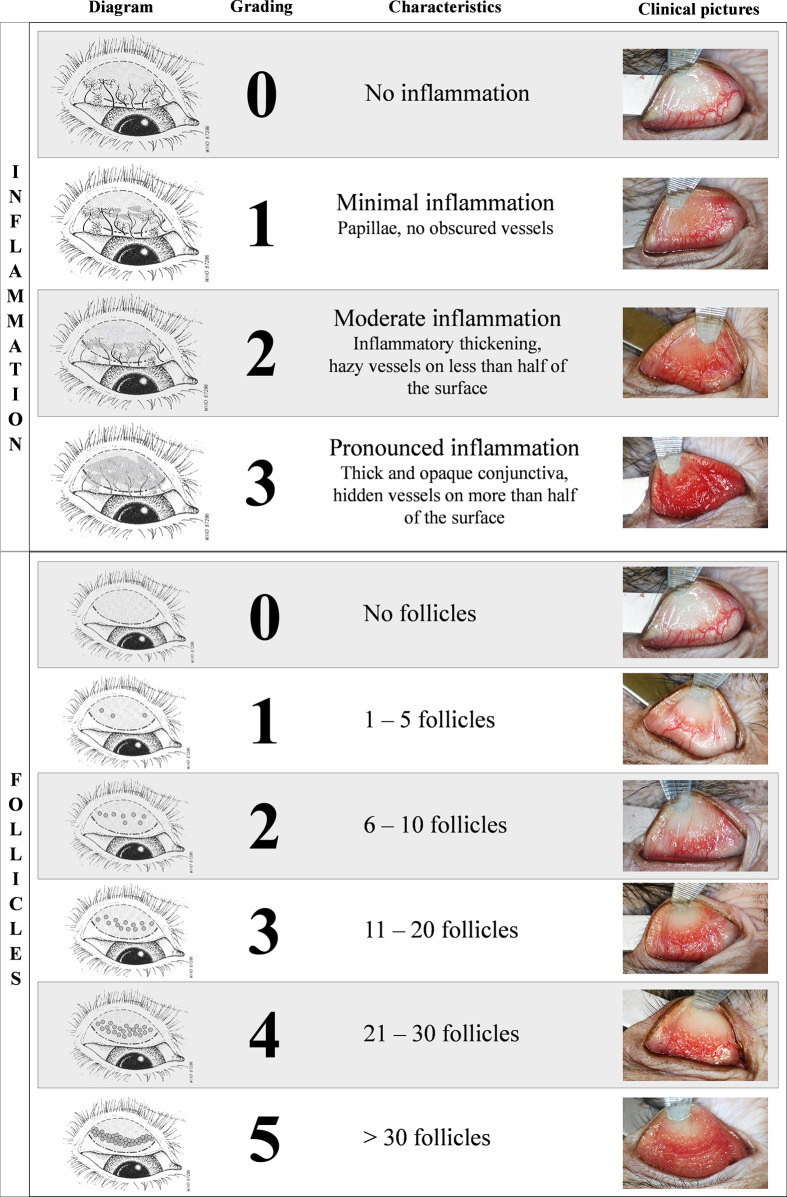
Clinical scoring of the conjunctival. Final score is calculated by adding the two components of the score: inflammation (graded on a scale of 0 to 3) and follicle (graded on a scale of 0 to 5), resulting in a maximum score of 8. Inflammation grading evaluated conjunctival edema, thickening, and the extent of hidden blood vessels. Follicle grading involves counting follicles in the central region of the upper tarsal conjunctiva. To obtain clear visibility, the upper eyelid was everted using a metal spatula and held in place with blunt forceps. Clinical photographs were captured with the animals under general anesthetic. The schematic diagram of the upper tarsal conjunctiva was adapted from [26] with the permission of the World Health Organization.

Conjunctival clinical scoring was performed with a customized clinical score using ocular surface photographs (**Fig 2**). This score was derived from the *WHO trachoma enhanced (FPC) grading scale* [26] and modified for compatibility with specific requirements of our model (**Fig 2**). The final score was calculated by adding the two components of the score: inflammation (graded on a scale of 0 to 3) and follicle (graded on a scale of 0 to 5), resulting in a maximum score of 8. Inflammation grading evaluated conjunctival edema, thickening, and the extent of hidden blood vessels. Follicle grading involved counting follicles in a predefined crescent-shaped zone of the upper tarsal conjunctiva (**S1 Fig**).

Ocular surface photographs were captured following a standardized protocol, as explained briefly: under general anesthesia with the macaques supine, the upper eyelids were gently everted using a spatula, then held in place using blunt forceps (Dutscher, 956507).

An EOS 80D camera (Canon), equipped with a Macro 100 mm Image stabilizer ultrasonic lens (Canon), and a Macro ring lite MR-14EXII circular flash (Canon), was mounted to a generic stand, facing down and positioned approximately 20cm above the eye of interest. Focus was set on the upper tarsal conjunctiva. Photographs were anonymized before scoring for inflammation grading and follicle counting by three independent observers masked to the experimental group (A.R., E.P., M.G.), including one ophthalmologist with sub-speciality training in human ocular surface infections (A.R.). Both eyes were scored independently by each observer. The photography images were scored as follows: i) if all three observers reached a consensus, the score was validated; ii) if two out of the three observers agreed, with the third observer differing by only 1 point on the clinical grading scale, the consensus score was retained and; iii) if all three observers disagreed or if one observer had a score differing by more than 1 point with the other 2 observers, a second round of masked grading was performed with three observers and the same criteria were applied. As both eyes were inoculated using the same protocol, the average bilateral score was used as the final score for each animal. Minor conjunctival inflammation was observed in healthy animals hence a total score of 2 or less was considered within normal limits.

### Bacterial load quantification

Conjunctival swabbing was performed using sterile flocked swabs (FLOQSwabs 519CS01, Copan, Italia) at the inferior conjunctival fornices instead of the superior fornices avoid conjunctival imprinting and not influence clinical scoring. The swabs then placed in 1 mL of Amies medium at room temperature and DNA was extracted using a QIAamp DNA Mini kit (Qiagen). Quantitative PCR was performed to assess the bacterial load using the Presto combined qualitative real-time CT/NG assay (Goffin Molecular Technologies, CG160100, and primer set previously described [27] CtPl+ 5’-TAGTAACTGCCACTTCATCA-3’ and CtP2 5’-TTCCCCTTGTAATTCGTTGC-3’) and using a CFX96 real-time thermocycler (Bio-Rad)). The bacterial load was calculated by the thermocycler software from a standard curve (obtained from 4x10^5^ to 4x10^0^ IFU/mL of bacterial stock strain B/Tunis-864 diluted in dPBS 1X) and expressed in IFU-equivalent copies,. The limit of detection was fixed at 4 equivalent IFU/mL and limit of quantification at 40 equivalent IFU/mL.

### Quantification of cytokines

Tears were collected with Dina strip Schirmer-Plus tear test kits (Coveto, France), with the test strip placed on the inferior conjunctival fornices until no more impregnation was observed (generally 2–3 minutes). Subsequently 50 μL of NaCl were added to the strip prior to centrifugation (19,000 x g for 20 minutes, Sigma 3-16PK) to extract the tears. Bead-based Luminex multiplex assay protocol (Milliplex Map, PRCYTOMAG-40K) was performed on 25 μL of diluted tears (completed with PBS to obtain a total of 70μL) to quantify cytokine concentrations (G-CSF, GM-CSF, IFNγ, IL-1β, IL-1RA, IL-2, IL-4, IL-5, IL-6, IL-8, IL-10, IL-12/23(p40), IL-13, IL-15, IL-17α, MCP-1, MIP-1β, MIP-1α, sCD40L, TGF-α, TNF-α, VEGF, and IL-18). The same protocol and panel was applied to 25 μL of undiluted serum samples for cytokine quantification.

### Conjunctival imprinting and flow cytometry

Local cellular immune infiltrates were evaluated with flow cytometric analysis of fluorescent antibody-labelled superficial conjunctival cell samples. Compresses were used to gently dry the upper tarsal conjunctiva. Imprints were harvested using 1.6 cm x 0.6 cm semi-oval pieces of nitrocellulose membrane (Supor PES Membrane disc filters, 0.2 μm 47 mm, ref-66234, Pall, Ann Arbor, Michigan, USA), which were applied to the upper tarsal conjunctiva with gentle pressure. The membrane was then carefully removed and placed into phosphate buffer saline (PBS). After 1 hour of gentle agitation at room temperature, cells that were adsorbed onto the membrane were eluted using a flushing technique, as described previously [28]. Eluted cells were blocked with 10% rat serum before labelling with a panel of fluorescently-conjugated antibodies: CD326 (EpCam)-PE (clone 1B7, Thermo Fischer 12-9326-42), HLA-DR-AF700 (clone L243 G46-6, BD 560743), CD45-BV786 (clone D058-1283, Becton Dickinson 563861), CD66-FITC (clone TET2, Miltenyi 130-116-663), CD14-BUV737 (clone M5E2, Becton Dickinson 612764), CD3-BUV395 (clone SP34-2, Becton Dickinson 564117), CD8-BUV805 (clone SK1, Becton Dickinson 612890), CD4-BV421 (clone L200, Becton Dickinson 562842), CD20-PE-Cy7 (clone 2H7, Becton Dickinson 560735), CD159a(NKG2A)-APC (clone Z199, Beckman Coulter A60797). The samples were then fixed with BD Cellfix (Becton Dickinson 340181), and acquisition was performed using a 5-laser 27-filter ZE5 flow cytometer (Bio-Rad). Data were analyzed using FlowJo software (v10.8.1, Becton Dickinson). The gating strategy was designed to quantify immune subsets such as neutrophils, monocytes, T lymphocytes, B lymphocytes, and natural killer (NK) cells (**S2 Fig**). Leucocyte counts (CD45+ cells) were normalized for 50,000 observed cells, and all other immune populations were normalized for 100 leucocytes (to obtain proportions of immune cell populations expressed as percentages).

### Serum and tear IgG and IgA quantification

Indirect quantitative ELISA was used to measure specific anti-Ct IgG and IgA antibodies in serum and tears. Positive IgG and IgA references were arbitrarily assigned a value of 5 ELISA-units/ml (AEU/ml). MaxiSorp plates (NUNC, Denmark) were coated overnight at 4°C with 4 μg/ml UV-inactivated B/Tunis-864 Ct elementary bodies in carbonate buffer pH 9.6. Plates were washed with PBS containing 0.05% Tween 20 and blocked with 2% bovine serum albumin (BSA) in PBS. Serum and tear samples were tested in duplicate, by 2-fold serial dilution in PBS with 1% BSA. IgA and IgG were detected by goat α-Human Fc IgA-Biotin conjugate and goat α-Human Fc IgG-Biotin conjugate (Southern Biotech, Birmingham, AL, USA) diluted 1:20,000 in PBS with 1% BSA and incubated for 1 hour at 37°C. This was followed by incubation for 40 minutes in a dark room with Poly-HRP40 (Fitzgerald, Acton, MA, USA) diluted 1:20,000 in PBS with 1% BSA. Detection substrate was TMB-PLUS (Kem-En-TEC, Denmark), the reaction was stopped with 0.5 M H_2_SO_4_ and absorbance was recorded at 450 nm (after subtraction of the background absorbance value measured at 620 nm). The IgG and IgA references were then used to establish a standard curve for the determination of titers in arbitrary ELISA units/ml (AEU/ml) based on a five-parameter logistic curve using the ‘drc’ package in R (version 4.3.1) [29].

For determination of total IgG and IgA in tears, the same ELISA protocol (MaxiSorp, Nunc) was used except that plates were coated with anti-human kappa and lambda light chain specific mouse antibodies (Southern Biotech) at 1:1 ratio diluted to 1 μg/ml in PBS overnight. Purified human IgG and IgA were used as standards (Sigma, St. Louis, MO, USA). Tear samples were tested by 4-fold serial dilution, and serial 2-fold dilutions of IgG and IgA standards were applied. Total IgG and IgA were calculated from the IgG and IgA standard curves based on a five-parameter logistic standard curve.

### Statistical analysis

Correlation between multiple parameters was assessed using the non-parametric Spearman correlation test, with a two-tailed p-value, performed using R software (version 4.3.1) [29]. For each combination of two parameters, this analysis assigned a correlation factor (r) from -1, a maximum negative correlation to 1, a maximum positive correlation, and the *p*-value. For all other statistical analyses, particularly to confirm significant differences during longitudinal observation, the Wilcoxon test and the Friedman test (with a two-stage linear procedure correction) were performed (Prism;GraphPad version 9.5.1). Full datasets are available on Dryad (https://doi.org/10.5061/dryad.69p8cz982) [30].

## Results

### Clinical manifestations of conjunctival infection

Two doses of inoculum were initially tested in a pilot study (**Fig 1**) to determine the lowest dose of Ct that resulted in disease in 100% of animals. From week 1 post- infection, both animals in both groups exhibited clinically-defined acute trachoma, characterized by the presence of combined conjunctival follicles and inflammation (**S3 Fig**). The two animals in the 10^4^ IFU/eye group initially had a clinical score of 2, which increased after exposure (3 at 1 week p.e. and 5 at 2 week p.e.). The clinical scores of all animals exposed to the bacteria (both 10^4^ and 10^5^ IFU/eye), remained stable after 2 weeks p.e., with scores of ≈ 5 for the 10^4^ IFU/eye exposure and ≈ 7 for the 10^5^ IFU/eye exposure. SPG buffer mock-inoculated controls maintained a clinical conjunctival score ≤ 2 for the follow-up period (**S3 Fig**). Subsequent experiments were conducted using the lower dose inoculum (10^4^ IFU) as it could elicit measurable disease in both animals.

**Fig 3:**
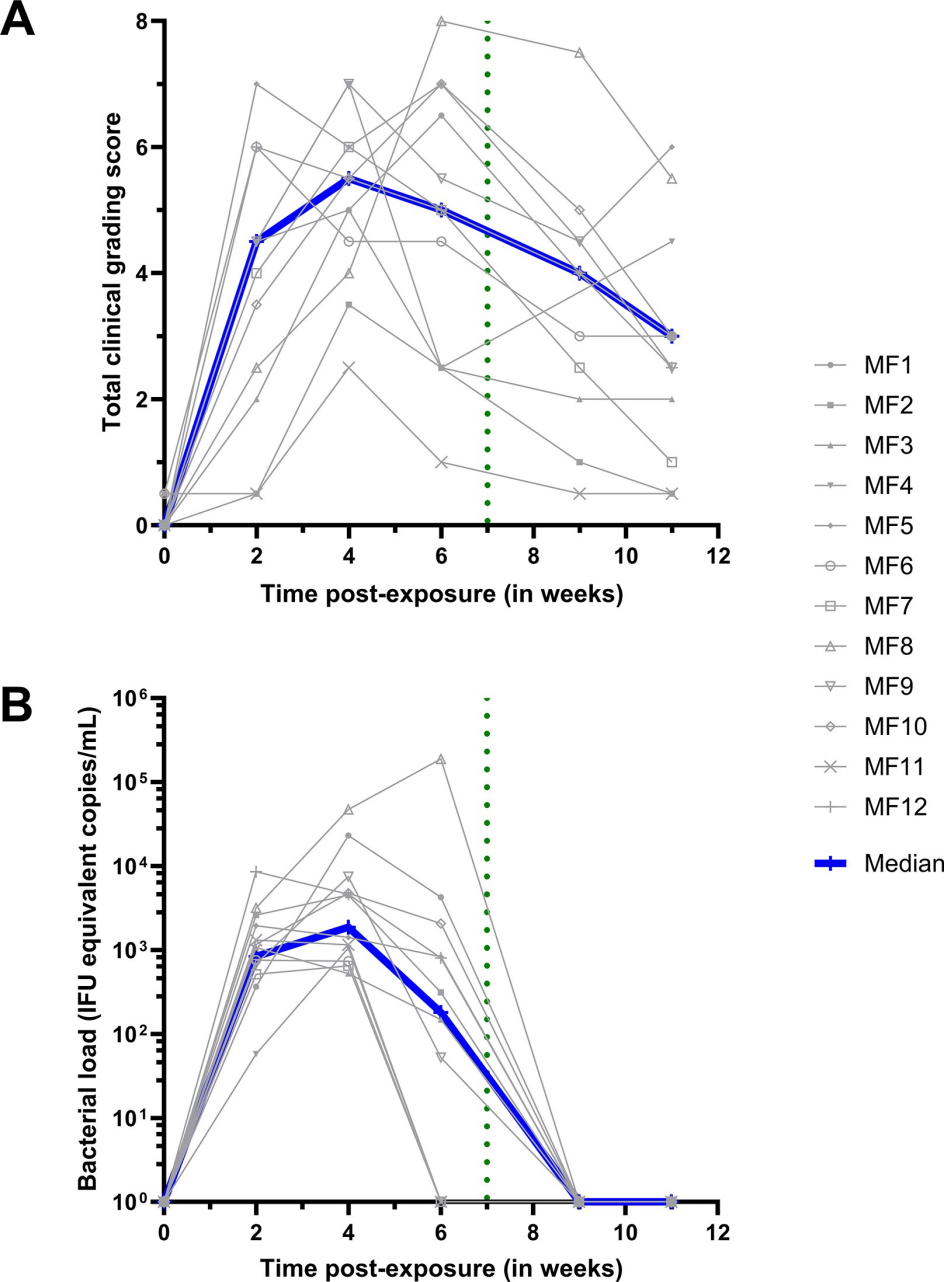
Ocular manifestations and bacterial load. The baseline was adjusted to week 0. All animals were infected with *C*. *trachomatis* (Ct) at week 0. The green dotted line represents the azithromycin treatment. The grey lines represent the mean bilateral values for each animal. (A) Ocular clinical grading scores were assessed using the *Clinical Grading Scale* (**Fig 2**) by three masked observers. The scores represent the average of both eyes and the three observers. The total clinical grading score for group 2 was determined by combining the inflammation and follicular scores (individual scores can be found in **S4 Fig**). The Friedman test, with a two-stage linear step-up procedure by Benjamini, Krieger, and Yekuteli, confirmed significant changes over the course of the study. (B) Bacterial load quantification is expressed in infection forming units (IFU) equivalent copies/mL. Experiments were performed for group 2 by conducting qPCR on DNA extracted from ocular fluids.

Clinical conjunctival scoring and bacterial load quantification were performed for up to 11 weeks in macaques (n = 12, MF1-12) infected with 10^4^ IFU of Ct (Group 2). At baseline, follicles (11/12, **S4A Fig**) and inflammation (10/12, **S4B Fig**) were almost completely absent. The outliers for follicles or inflammation were mild (scoring 1 and 0.5, for inflammation by MF5 and MF6, respectively, and a 0.5 follicle score for MF2). Follicular conjunctivitis was observed in all animals from 2 weeks post-exposure (p.e.) (12/12, *p*<0.0001), with 8/12 also demonstrating inflammation (median score = 2) (**S4A and S4B Fig**). Clinical scores peaked at 4 weeks p.e. and then steadily decreased (**Fig 3A**). Although at 6 weeks p.e., higher than the average clinical scores were recorded for almost half of the group (n = 5 macaques) for the remainder of the study. At week 6, the inflammation score decreased for 5/12 animals. However, median clinical scoring decreased *post*-azithromycin treatment (p.t.), as inflammation decreased in 10/12 macaques (**S4B Fig**).

All other clinical parameters, including weight, temperature, and complete blood count, remained stable throughout the study, except for blood neutrophils, which increased following exposure to Ct (**S5 Fig**).

### A robust conjunctival *Chlamydia trachomatis* infection

Conjunctival infection was monitored by changes in bacterial load, characterized by Ct-specific genomic qPCR (**Fig 3B**). All animals harbored Ct infection, which was detected at the first time-point of testing (2 weeks p.e.). Peak bacterial load was measured at 4 weeks p.e., and decreased in all macaques except one (MF8), in which kept increasing until after p.t.. In 4/12 macaques, the bacterial load decreased to undetectable levels at 6 weeks p.e. In the 8 remaining macaques the bacterial load decreased to undetectable levels by week 9, which was 2 weeks p.t. (**Fig 3B**).

These outcomes demonstrate robust conjunctival Ct infection of all macaques that produced scorable clinical disease.

### Assessment of the conjunctival immune response

#### Immune cell populations at the ocular surface

**Fig 4:**
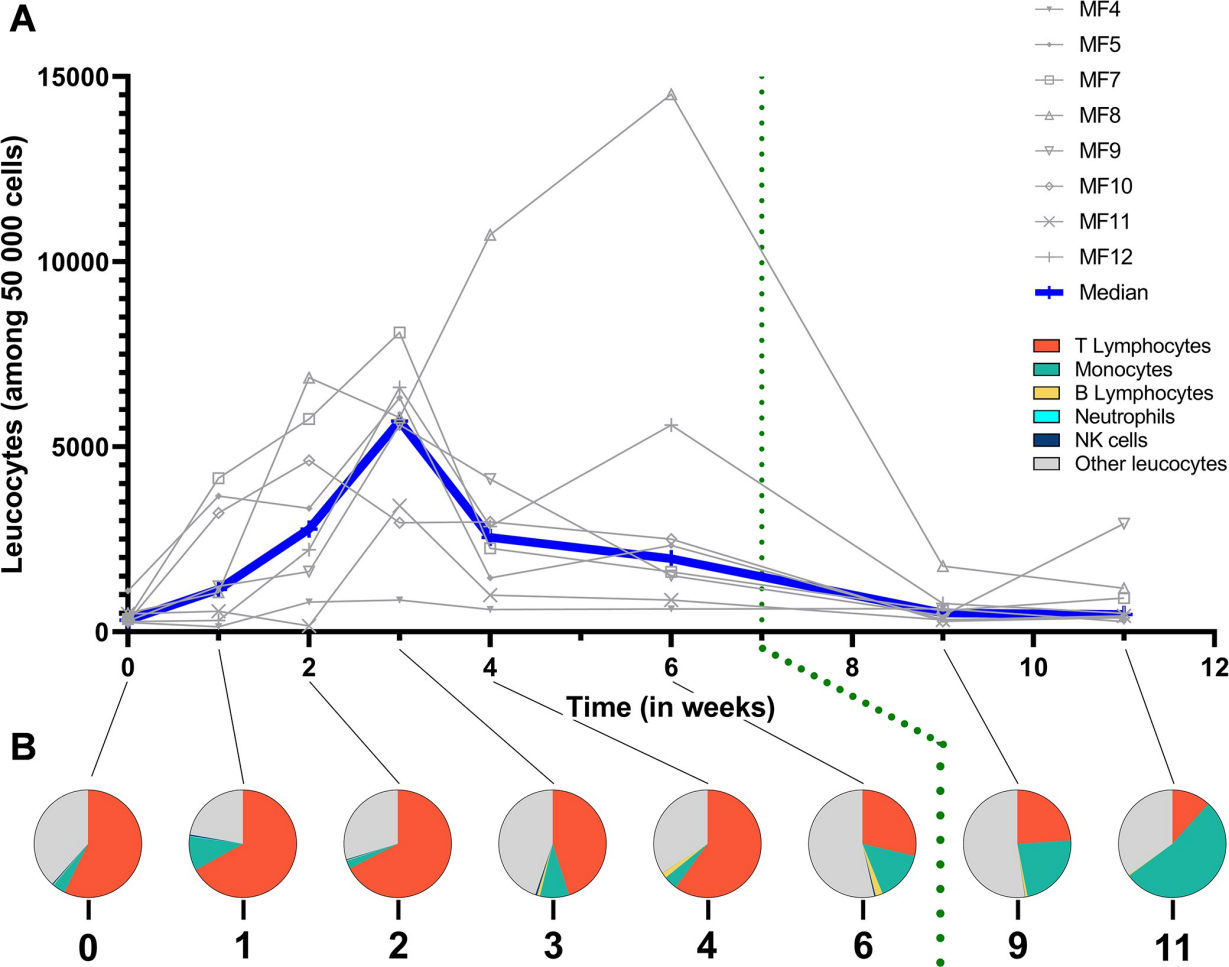
Multiparametric flow cytometry of superficial conjunctival cells. (A) Leucocytes count normalized for 50,000 cells. (B) Specific proportions of the immune population normalized for 100 leucocytes. The baseline was adjusted to week 0. All animals were infected with *C*. *trachomatis* at week 0. The green dotted line represents azithromycin treatment. Other leucocytes category represents non-identified immune cells. The Friedman test, with a two-stage linear step-up procedure by Benjamini, Krieger, and Yekuteli, confirmed significant changes over the course of the study.

Flow cytometry was performed on conjunctival cells sampled during the course of infection to assess the major constituents of the local immune response. Leucocyte numbers (CD45^+^ cells normalized to 50,000 observed cells) increased at 1 week p.e., and generally peaked at 3 weeks p.e. (*p* = 0.0078 baseline vs week 3 p.e.) (**Fig 4A**). The most common leucocytes detected by sampling were T cells, monocytes, and “other leucocytes”, defined as CD45^+^ cells negative for all other immune markers (**S2 Fig**). During the first 2 weeks p.e., the proportion of T cells increased slightly from baseline (57 ± 8% to 67 ± 9%, p = 0.1210), and remained stable (**Fig 4B**). Between weeks 3 and 4, T cells proportions fluctuated and by week 6 the proportions had decreased. Interestingly, between weeks 3 and 6 p.e., significant proportions of B cells (CD20^+^ HLADR^+^-double positive CD45^+^ cells) were detected with the highest proportion at 6 weeks p.e. (2.14 ± 0.57%, *p* = 0.0156 for week 6 vs week 1 p.e.) (**Fig 4B**). The proportions of monocytes fluctuated from week 1 to week 4 p.e. but did significantly differ from baseline (*p* = 0.8750). Subsequently their proportion among leucocytes steadily increased from 6 weeks p.e. and peaked at 11 weeks p.e. (53 ± 9% of all immune cells, *p* = 0.0078 for week 11 (final time tested) vs week 1 p.e.). Negligible proportions of neutrophils (CD66^+^ cells) and natural killer (NK, CD159a-NKG2A^+^ cells) cells were observed throughout the study period (less than 1% of immune cells).

These data demonstrate the local fluctuations of key immune subsets responding to acute Ct infection and treatment.

#### Dynamics of cytokine secretion in response to Ct at the conjunctiva

**Fig 5:**
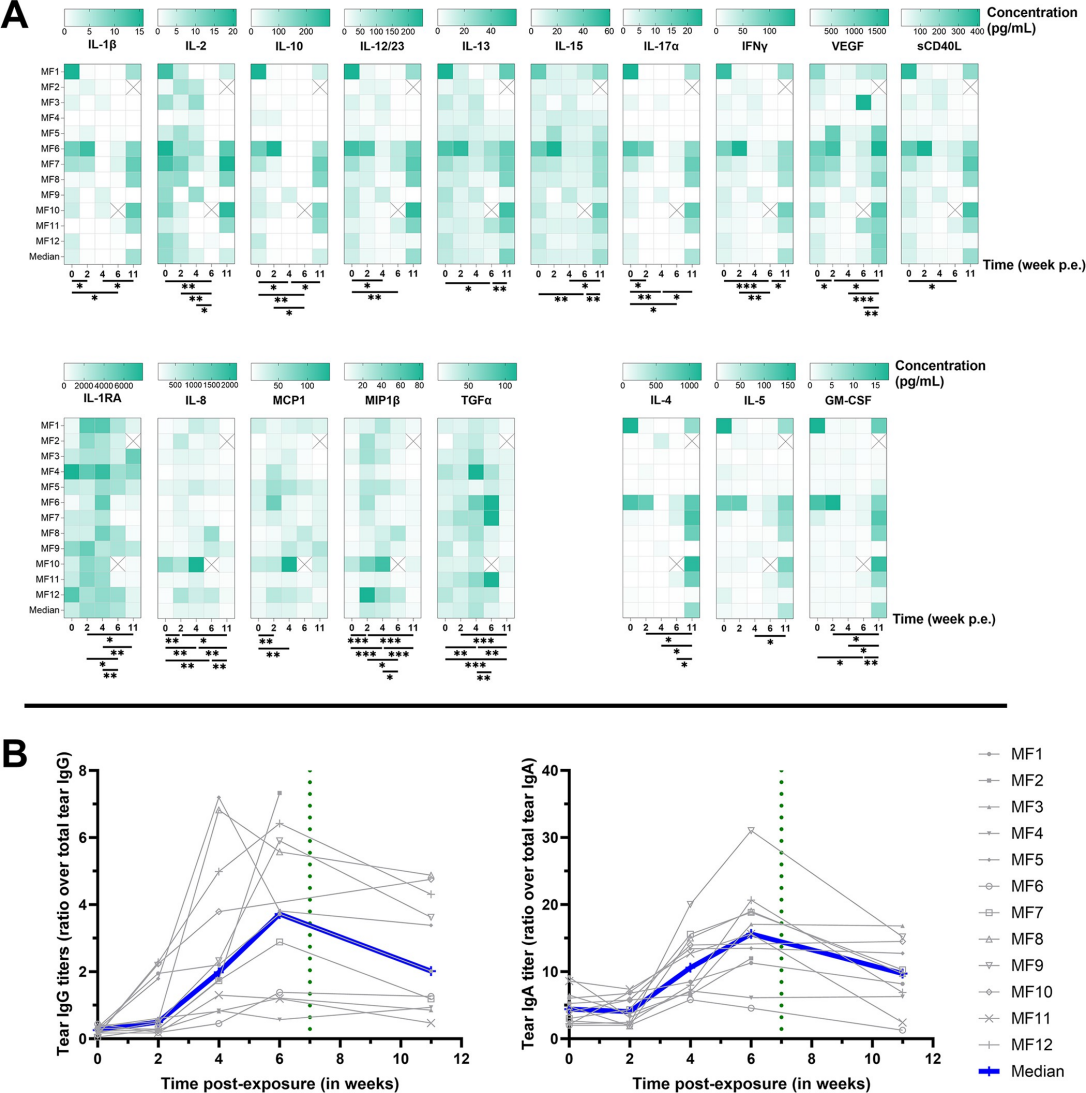
Cytokines (A) and IgG and IgGA secretion (B) in tears. The baseline was adjusted to week 0. The Friedman test, with a two-stage linear step-up procedure by Benjamini, Krieger, and Yekuteli, was utilized to confirm significant changes over the course of the study. **** *p*<0.0001, *** 0.0001<*p*<0.001, ** 0.001<*p*<0.01, * 0.01<*p*<0.1. The crossed boxes represent missing data. Cytokine quantification was performed with Luminex on tears. IgG and IgA quantification performed by specific ELISA assay on tears. Results are shown as a ratio of Ct-specific tear Ig over total tear Ig.

Ocular surface cytokines were quantified using Luminex to further investigate local immune responses (**Fig 5A**). At baseline (before infection), most cytokines were consistently detected in the conjunctiva of macaques. Some cytokine concentrations (TNF-α, MIP-1α, IL-6, IL-18, and G-CSF) did not significantly change after exposure to Ct (**S6 Fig**). Cytokines VEGF, IL-12/23, IL-13, IL-17α, IL-10, IFN-y, IL-2, IL-15, sCD40L and IL-1β decreased between weeks 4 or 6, or during both timepoints, but rebounded at week 11 p.e., to levels higher than baseline, and this effect was observed in more macaques. The detection pattern differed for TGF-α, MIP-1β, IL-8, MCP-1, and IL-1RA, which all increased at some point between weeks 2 to 6 p.e. (*p* = 0.0239, 0.0003, 0.0068, 0.0239, and 0.0049 at peak respectively) and subsided by week 11. Finally, a fourth pattern was observed with cytokines IL-4, IL-5 and GM-CSF remaining low throughout the course of the experimental period, then dramatically increasing only at week 11. These results demonstrate 4 different conjunctival cytokine response patterns to Ct as follows: no significant change, increase or decrease in correlation to bacterial load, or increase following treatment once the bacterial load was cleared.

#### Increased local IgA and IgG in response to Ct exposure at the conjunctiva

IgA and IgG specific to Ct elementary bodies were quantified from tears to determine the local adaptive humoral response to conjunctival Ct exposure. IgG increased comparably at 2 weeks p.e. (*p* = 0.021), then both IgG and IgA peaked between 4 and 6 weeks p.e. (*p* = 0.001 and 0.002 between weeks 0 and 6 for IgG and IgA respectively), and then decreased at 11 weeks p.e. (*p* = 0.0098 and 0.0039 for IgG and IgA respectively) (**Fig 5B**). These data demonstrate that local adaptive humoral responses developed over the first 6 weeks p.e.

### Systemic response to conjunctival Ct exposure

**Fig 6:**
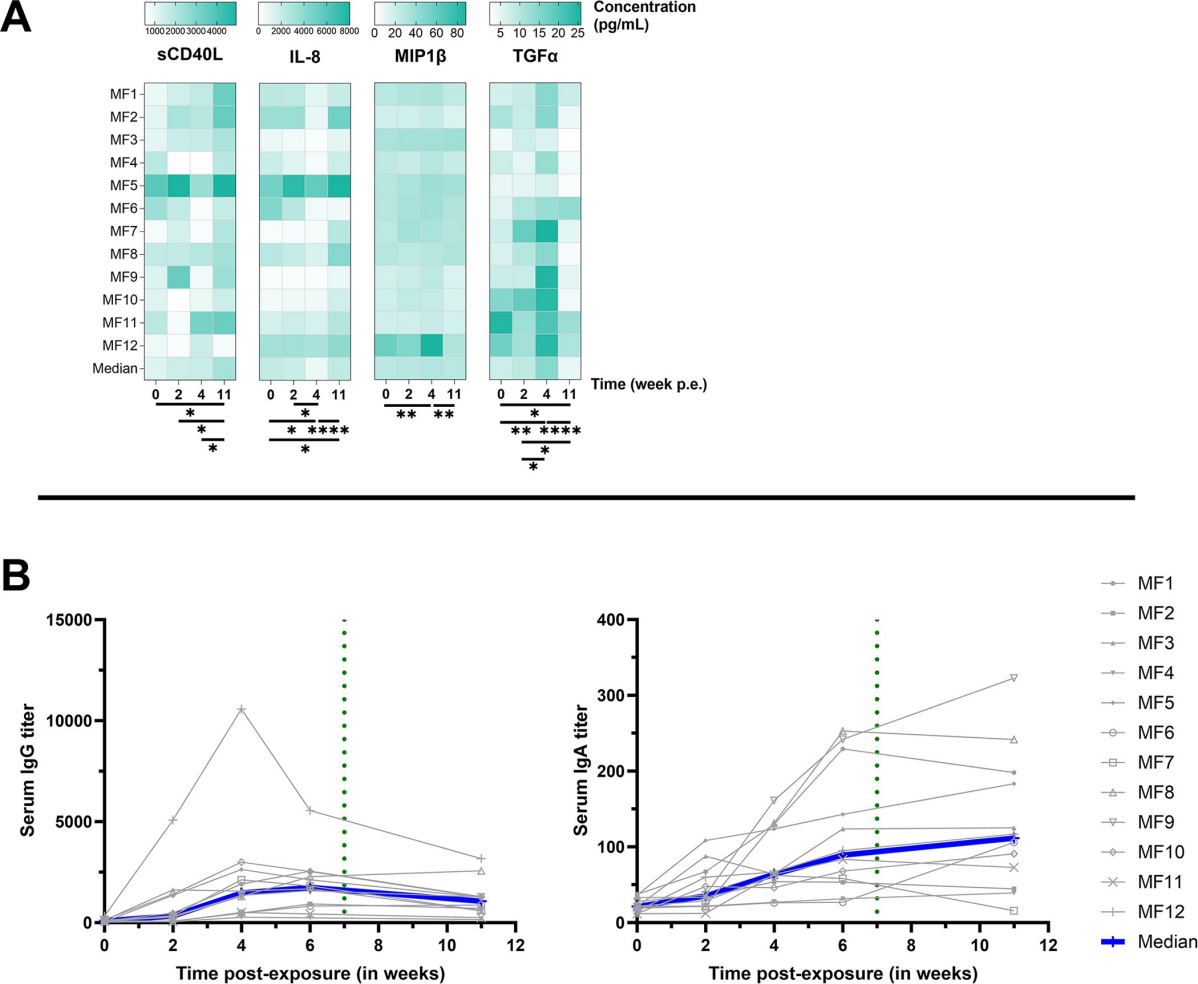
Cytokines (A) and Ig secretion (B) in serum. The baseline was adjusted to week 0. Cytokine quantification performed by Luminex on serum; IgG and IgA quantification performed by specific ELISA assay on serum. The Friedman test, with a two-stage linear step-up procedure by Benjamini, Krieger, and Yekuteli was utilized to confirm significant changes over the course of the study. **** p<0.0001, *** 0.0001<p<0.001, ** 0.001<p<0.01, * 0.01<p<0.1.

Serum cytokines and specific IgG and IgA were measured from sampled serum. Interestingly, the pattern of TGF-α detection in serum matched the fluctuations observed in tears, increasing to peak at 4 weeks p.e. (*p* = 0.004 between weeks 0 and 4), then subsiding by week 11 (*p*<0.0001 between weeks 4 and 11, **Fig 6A**). In contrast, sCD40L continued to increase in serum at each time-point tested, to peak at week 11 p.e. (*p* = 0.01 between weeks 0 and 11). IL-8 in serum decreased by 4 weeks p.e. (*p* = 0.04 between weeks 0 and 4), only to rebound to higher levels than baseline at week 11 (the opposite of the pattern in tears, *p* = 0.03 and *p*<0.0001 between weeks 0 and 1, and 4 and 11 respectively). MIP-1β detection pattern in serum mimicked the pattern in tears, increasing by week 4 (*p* = 0.005) and subsiding by week 11 (*p* = 0.003 between weeks 4 and 11, **Fig 6A**). Those results suggest that, while limited, sCD40L, TGF-α, and MIP-1β secreted by different immune cells may play a role in early systemic response to Ct exposure, while IL-8 could be implicated in the later phase of infection.

Additionally, circulating Ct-specific IgA and IgG quantification was performed on serum samples (**Fig 6B**). Increases in IgG were detected in 7/12 animals p.e., peaking between weeks 4 and 6 (*p*<0.0001 between weeks 0 and 4 and between weeks 0 and 6) and slightly decreasing by week 11 (*p* = 0.04 between weeks 6 and 11). Ct-specific IgA increased in 10/12 animals from 2 weeks p.e., but unlike IgG, continued to rise between week 6 to week 11, although this increase was not significant (*p* = 0.52). These results demonstrate that conjunctival Ct exposure elicits systemic circulation of Ct-specific IgA and IgG, which remained elevated to the latest time-point tested.

### Biomarker signature associated with clinical scoring and bacterial load

**Fig 7:**
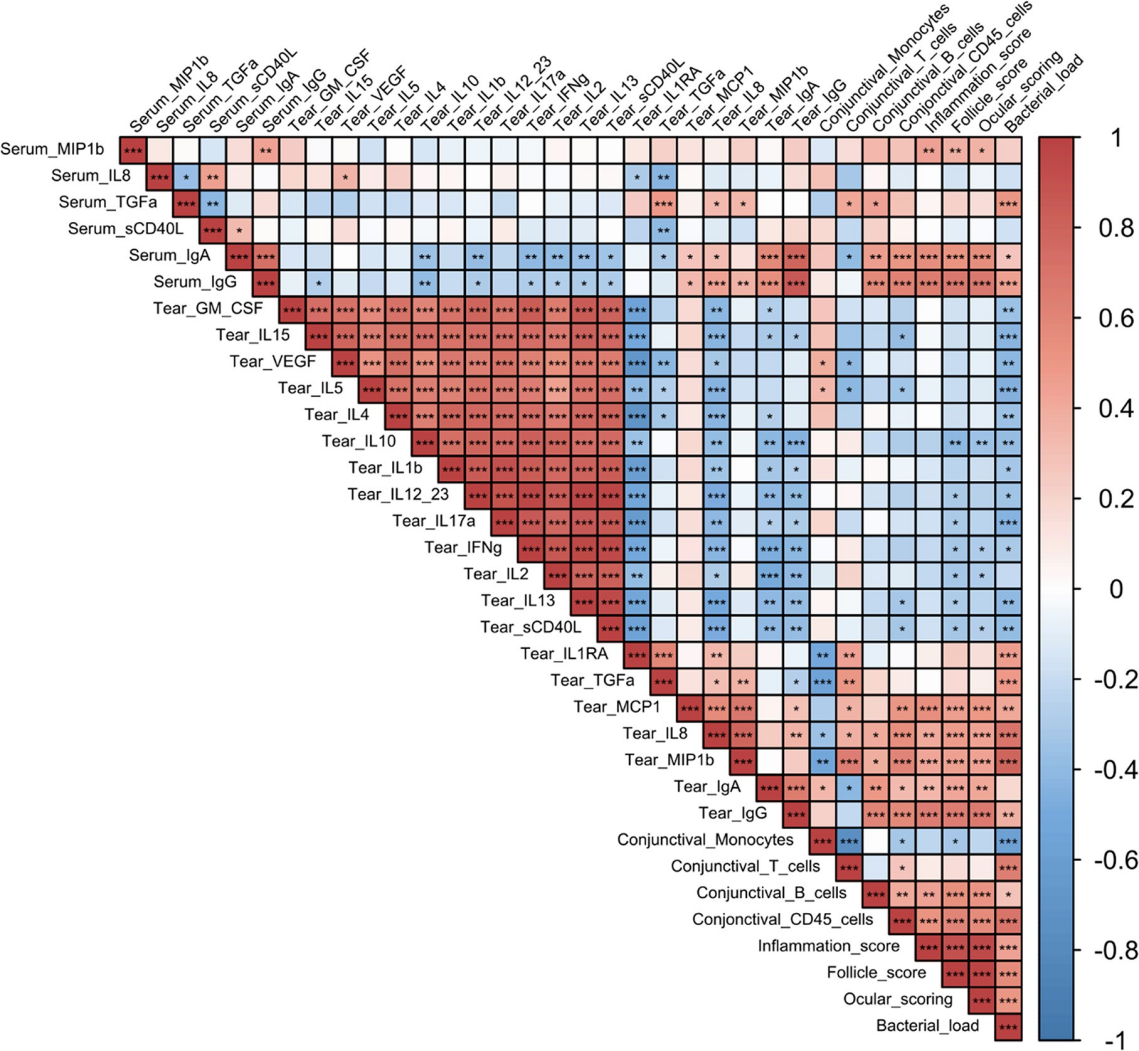
Correlation matrix of all the different parameters measured in group 2 for all time-points. Correlation was performed with a non-parametric Spearman correlation test (performed with R software) from -1, maximum negative correlation, to 1, maximum positive correlation. *** *p*<0.001, ** 0.001<*p*<0.01, * 0.01<*p*<0.05.

All of the parameters analyzed above, at all time-points, were assessed for correlations using the Spearman correlation test (**Fig 7**). This combined analysis uncovered 2 distinct signatures: a positive or a negative correlation with bacterial load.

Increases in clinical signs (inflammation, follicles and total clinical signs) correlated with each other, as well as with bacterial load (*p*<0.0001, for each condition vs bacterial load). Total conjunctival leucocyte increases (CD45^+^ cells immune infiltrate) were correlated with increases in clinical signs and bacterial loads (*p*<0.0001, vs both clinical signs and bacterial load). B cell increases correlated with bacterial load changes (p = 0.048), and increases in both tear and serum IgG (*p* = 0.004 and *p* = 0.0003, for tear and serum respectively). Serum IgA and IgG increases were correlated with increased bacterial loads, while in the tears, only IgG increases were significantly correlated to bacterial load (*p* = 0.034) and the positive correlation with tear IgA was not significant (*p = 0*.*1685*). Among cytokines, tear MIP1β, MCP-1, IL-8, TGFα, and IL-1RA were positively correlated with bacterial load (*p*<0.0001, *p* = 0.0011, *p*<0.0001, *p* = 0.0001, and *p* = 0.0002, respectively). In contrast, tear cytokines GM-CSF, to sCD40L (GM-CSF, IL-15, VEGF, IL-5, IL-4, IL-10, IL-1β, IL-12/23, IL-17α, IFN-γ, IL-13, and sCD40L) all correlated with each other, but were all negatively correlated with bacterial load and clinical signs. Taken together, this combined analysis identifies a biomarker signature that is indicative of Ct bacterial load and clinical signs associated with Ct, while a separate signature was associated with decreased bacterial loads and clinical signs.

## Discussion

### An accurate NHP model of trachoma mimicking clinical features of human disease

Similar to previously reported trachoma NHP models [3,12,13], our model faithfully reproduces the clinical features of acute trachoma observed in human patients in endemic populations. In humans, conjunctival Ct infection is followed by follicular conjunctivitis and conjunctival inflammation, which develop 1–2 weeks p.e. [5]. In our study, Ct inoculation elicited clinical signs in all macaques within the same time frame (**Fig 3B**), and their increasing severity was accompanied by increases in conjunctival bacterial loads [4]. In humans, Chlamydial infections are typically cleared within 3 to 8 weeks, but clinical signs of inflammation can persist for 6 to 18 weeks [24], which concurs with the experimental findings in our model, since follicles persisted out to 11 weeks after bacterial exposure, i.e. 2 to 5 weeks after bacterial clearance. The clinical findings in our model are comparable to guinea pig models of inclusion conjunctivitis obtained with a single exposure which develop clinical manifestations at 7 days p.e.. However, macaques allow for a more comprehensive clinical characterization of lesions due to the larger size of the NHP eye and conjunctiva compared guinea pigs [31,32].

Given the ongoing burden of trachoma as a public health issue [1,2], it is crucial to have an accurate model for studying the disease [9,11]. Previous studies have used cynomolgus monkeys to reproduce trachoma in NHP. Taylor *et al* successfully induced conjunctival fibrosis, replicating the full spectrum of the disease [3]. The monkeys were infected on a weekly basis for 52 weeks, highlighting the importance of chronic exposure to Ct for advanced stage disease. Kari *et al* performed dual Ct exposure, also in cynomolgus monkeys, to test a candidate trachoma vaccine. The second exposure was administered three months after spontaneous clearance of the first inoculation. However, late fibrotic stages of disease were not observed [33].

In our model, a single Ct exposure was employed to characterize clinical and immune makers of acute infection, with the ultimate goal of assessing the efficacy of new vaccine candidates in the future. The novelty of our model is the development of an optimized clinical grading score (**Fig 2)** [26,34,35], allowing precise assessment of the kinetics of the clinical disease (**Fig 2**), combined with multimodal exploration of local and systemic immune responses. Optimization of the dose and the observation of clinical signs, allowed for precise assessment of the kinetics of clinical disease development. Clinical signs developed in similar manner to human disease. Incorporating non-invasive sampling techniques such as conjunctival imprints proved to be highly valuable in obtaining consistent measurements and establishing novel tools to study kinetics of local immune populations. Furthermore, the analysis of tears encompassed a deeper understanding of the local inflammatory immune landscape in our model. Indeed, our longitudinal *in vivo* data showed similar results to one-time sampling from different trachoma stages in endemic area human cohorts, validating this experimental procedure to faithfully mimic clinical features of human disease [36].

### Two phase immune response: before and after treatment

#### Infectious phase response

Immune infiltrates peaked from weeks 2 to 4 p.e., with a major proportion consisting of T cells. The immune response associated to bacterial load observed in the first 7 weeks was also associated with conjunctival T lymphocytes (*r = 0*.*63*, *p < 0*.*0001*) and T cell survival factors (IL-2 and IL-15) were frequently detected during this period. In humans, T cells seem to play a major role in disease pathogenesis but are also associated with clearance of Ct infection, driven by CD4 T cell-derived IFN-γ [5]. Interestingly in our model, conjunctival T cell numbers did not correlate with T-cell associated cytokines and instead IL-10, IL-2, IL-4, IL-5, IL-17α, and IFN-γ all peaked at week 11 p.e. This finding could be explained by the fact that cytokines typically associated with T cells might also be secreted by other immune cell types. The early conjunctival T cell spike was instead associated with cytokines excreted by epithelial cells (IL-1RA, IL-8) and macrophages (MCP-1, MIP1β). Analysis of conjunctival swabs from patients indicate a strong correlation between Th1 pro-inflammatory cytokines (IL-1β and TNFα) in acute trachoma and Ct bacterial load [37]. In contrast, Th1 cytokines evaluated in our panel (IL-1β, TNF-α, and GM-CSF) did not significantly increased p.e. [36].

In trachoma patients, increased cytokines levels have also been reported for, IL-10 (associated with anti-inflammatory response), CXCL8 (IL-8, involved in recruiting granulocytes and facilitating phagocytosis), and CCL2 (MCP-1, involved in attracting monocytes, lymphocytes, and basophils, as well as promoting macrophage differentiation) [37]. These findings align with our results where IL-8 and MCP-1 significantly increased after Ct exposure. The importance of these variations in cytokine levels lies primarily in the search for an early indicator of trachoma. Although they are insufficient to identify a specific marker, these results indicate that we can detect some variation. With further studies, we hope to identify a reliable early-stage marker for trachoma.

In animal models, neutrophils have also been shown to play a central role in the response to acute trachoma. Lacy *et al* reported their involvement in T cell recruitment (both CD4^+^ and CD8^+^) by using neutrophil depletion in a guinea pig model of *Chlamydia cavia* infection [17]. The recruitment of CD4^+^ and CD8^+^ T cells by the influx of neutrophils was a beneficial immune phenomenon that helped to clear the infection [17]. Although this T cell recruitment reduces the bacterial burden, it may simultaneously exacerbate ocular symptoms (follicles and inflammation) [19]. This observation has also been reported in a murine model of Ct vaginal infection with concomitant neutrophil depletion [19]. Lacy *et al* also reported a link between neutrophil depletion and increased IgA titers [17]. This observation could mean that in the absence of neutrophils, while clinical signs are reduced, local IgA increases can occur due to a local increase in B lymphocytes [17]. Similarly, we observed a tendency toward decreased ocular signs around 6 weeks p.e. corresponding to a peak in specific anti-Ct IgA in tears, concomitant with a higher proportion of B cells. Transcriptome studies in humans with active trachoma inferred a prominent role of the innate immune response, notably neutrophils. Natividad *et al* identified immune infiltrates with immune populations such as T cells, B cells, and a predominance of innate immunity (both NK cells and neutrophils) [38].

In our study, while we did not detect significant neutrophil infiltrates (due to technical limitations), neutrophils in blood increased during the peak of infection (between 2 and 4 weeks p.e.). However, we found significant T and B cell conjunctival infiltrates (**Fig 4**) that corresponded to follicular lesions, which is consistent with human disease [20,38] and other animal models [12,17]. We found, a positive correlation between clinical signs and B lymphocytes infiltrate (r coefficient = 0.52, *p* = 0.00032).

A study in 1977 on immunoglobulins in the tears of trachoma patients reported a lower quantity of IgA in trachoma cases compared to healthy patients (without significant differences between trachoma phases) [39]. However, Taylor *et al* used a Cynomolgus model of trachoma and a different Ct strain (E/Bour), found a delayed increase in tear IgA after conjunctival Ct exposure, and persistence of systemic IgG [40] which is consistent with our findings.

#### Convalescent phase response

The convalescent phase was defined as the period after treatment from 7 weeks p.e. This phase was characterized by an absence of Ct due to either spontaneous clearance (for 4/12 macaques) or the effect of azithromycin treatment. Bacterial clearance coincided with a reduction in conjunctival inflammation, although the presence of follicles persisted, a dissociated pattern that has also been observed in humans [41]. Studies conducted on human cohorts at different stages of trachoma revealed an association between chronic trachoma and the local presence (in mucosal sponges) of IL-1RA (an antagonist of the pro-inflammatory IL-1), as well as IL-4 and IL-13 (associated with Th2 response) [37].

The decreased population of local leucocytes at 9 and 11 weeks p.e. could be a result of the higher quantity of IL-1RA at earlier time-points, that may have had an anti-inflammatory effect. At these later time-points, although we observed a reduction in local leucocyte populations, we also found a higher proportion of monocytes, the cell type mainly responsible for IL-1RA secretion.

In a study of 470 Tanzanian children, transcriptome analysis of ocular fluid samples revealed a correlation between acute trachoma and elevated expression of IL-17A [41]. The authors concluded that IL-17A and Th17 responses play a central pro-inflammatory role in trachoma [20]. A transcriptomic study on an Ethiopian cohort of trachomatous patients also suggested a central role of other pro-inflammatory cytokines (including IL-1B, CXCL5 or S100A7) in chronic trachoma [42]. In the present study, the increase in IL-17A was delayed, (week 11 p.e.), along with elevated levels of other pro-inflammatory (VEGF, IL-1B, IL-15, and GM-CSF). Some anti-inflammatory cytokines (IL-10, IL-4, and IL-5) were also elevated but with a delay, suggesting that a balance between both anti- and pro-inflammatory cytokines is needed to achieve disease resolution. Those results could also be considered in the context of the infectious dose used. A study on guinea pigs comparing different infectious doses showed an increase in T cell response for higher infectious doses [43], which may be similar in monkeys. Since the infectious dose in human trachoma is unknown, further exploration of the immune response with different Ct infectious doses could potentially yield results more reflective of the human immune response.

A longitudinal study of a Tanzanian cohort of children identified an association between progressive trachomatous scarring and elevated levels of IL-23 which inidicate the importance of a Th17 response (through the analysis of IL-23A and PDGFB) [44]. Our observations of the late increase in both IL-12/23 and IL-17A concur with these findings.

In our study there was a decrease in total leucocyte counts from week 3 p.e., that dropped sharply at weeks 4 and 6. During this decrease, the proportion of monocytes increased significantly then subsided p.t.. These observations suggest that local monocytes are characteristic of the continuance of clinical signs of inflammation once bacterial infection resolves. Diminution of the proportion of T cells in the model infection is also consistent with observations in the active stages of trachoma in humans. It has been shown that T cells, particularly CD4^+^ T cells, and the Th2 response correlate with scarring trachoma both locally [37,45] and systemically [46].

### Limitations

While our results enhance our understanding of the pathogenic mechanisms of trachoma, a clear limitation of this study is its design as an acute model, which does not replicate the multiple recurrent infections characteristics of the human disease [40].

The choice of the Ct strain used in this study was based on previous work by our team and our partners. However, investigating other Ct strains for trachoma could also be beneficial for future research.

The absence of a control group not treated with azithromycin represents another limitation of this study. This decision was primarily based on adherence to the 3R guidelines and to align with a concurrent study not included here. The lack of a control group precludes definitive conclusions about the effect of the treatment.

We acknowledge that the indifferent use of male and female monkeys may be questionable, as scarring and blinding trachoma is more frequent in females. However, a significant body of work suggests that repeated exposure through childcare, is likely the primary reason for the greater impact of active disease (and this is not applicable in the conditions of our model). Furthermore, research in endemic countries has not demonstrated that women or girls are more biologically susceptible to the consequences of infection with *C*. *trachomatis* [5].

The use of conjunctival imprints offers several advantages, such as being non-invasive and allowing for repeated sampling over a short period. However, this technique for harvesting cells can affect their viability. Certain sensitive cells such as neutrophils, may lose their viability during the elution step of the protocol [47]. Therefore, the lack of observed neutrophils by conjunctival imprint cytometry is likely due to the technical process rather than their absence. Additionally, imprints will likely capture only the cells present on the conjunctival surface, or those exposed to this sampling site in the context of a lesion and cells in the dermal conjunctival layers are likely not captured by this method. Sampling of the conjunctival surface may induce a bias in the type of cells sampled by imprints (bias that would be in favor of immune component of follicles and desquamating cells).

The non-identification of a significant proportion of leucocytes (labeled as “other leucocytes”) could be due to missing markers in our panel (such as markers for dendritic cells or granulocyte populations), technical difficulties (dead cells, non-leucocytes expressing CD45 antigen), or could imply non-specific binding of antibodies [48]. The panel of antibodies used in our flow cytometric analysis allows general identification of immune cell populations, but not identification of their specific subpopulations that have a wide spectra of functional characteristics (including among others: Treg/Th1/Th2/Th17 lymphocytes and M1/M2 macrophages). Furthermore, with the applied gating strategy, the possibility of unintentional misclassification of the same cells exists (for instance, both monocytes and macrophages could express CD14, and some monocytes could be CD14dim/-).

Therefore, optimizing our flow cytometry panel could improve the range of cell populations identified with additional information on the functional characteristics, especially for T cells and macrophages. Notably, our current model has laid the groundwork for the characterization of the local immune response; however, in order to understand the specific adaptive immune response to ocular infection, a more comprehensive analysis of specific T cell response is required. As shown in other experimental studies on macaques, CD8+ T cells have a role in protective immunity to Ct infection [12]. While we were able to measure variations in conjunctival CD4+ and CD8+, further studies are warranted to more precisely define their implication in the immune response to trachoma. Furthermore, comprehensive insights into the activation status and subtypes of these T cells, including additional markers such as IFNγ, CD69, or CD25, are imperative to fully evaluate their role in the immune response to Ct.

To summarize, our NHP model of acute trachoma adequately reproduces hallmarks of acute human disease. Most of the changes in immune effectors were observed at the local level, with an influx of T lymphocytes at the peak of infection and the persistence of monocytes with clinical signs in the absence of infection. This acute model could be further developed to investigate a chronic trachoma model by repeated Ct inoculations to induce conjunctival fibrosis, for pre-clinical assessment of therapeutic strategies and characterization of immune responses. This infection model may also be of importance in studies of immune-modulation treatment and in the identification of non-invasive correlates of protection for use in human vaccine trials.

## Supporting information

S1 Fig Follicles counting area in the superior tarsal conjunctiva. Follicle counting area is shown in red at the interface of the tarsal part (blue) and the orbital part (pink) of the palpebral conjunctiva.
(TIF)

S2 Fig Gating strategy of pseudo-color plots windows performed using FlowJo software and applied to cells eluted from conjunctival imprints. This strategy was used to assess the following ocular surface cell immune populations: leukocytes (CD45^+^), neutrophils (CD66^+^), monocytes (CD14^+^), T cells (CD3^+^), CD4 T cells (CD3^+^, then CD4^+^/CD8^-^), CD8 T cells (CD3^+^, then CD4^-^/CD8^+^), natural killer cells (CD3^-^/CD8^+^, then NKG2A^+^), B cells (CD20^+^ and HLA-DR^+^). This strategy is applied to conjunctival imprints’ cells’ flow cytometry data.
(TIF)

S3 Fig Total Conjunctival clinical scores of Group 1. Each line represent the mean of 4 clinical scores (one score per eyes and two animals per subgroup) of animals respectively exposed to 104 IFU of Ct (blue line), to 105 IFU of Ct (purple line), and only to the SPG buffer (grey line).
(TIF)

S4 Fig Conjunctival clinical scoring results for follicule score (A) and inflammation (B) in Group 2
(TIF)

S5 Fig Blood neutrophil count in group 2. (performed at each time-point as part of a complete blood count.
(TIF)

S6 Fig Cytokines quantification without statistically significant changes quantified on tears (A) and serum (B). The crossed boxes represent missing data.
(TIF)
