# Supplementary figures and images for: Multimodal mucosal and systemic immune characterization of a non-human primate trachoma model highlights the critical role of local immunity during acute phase disease
Source: PLoS Negl Trop Dis. 2024 Aug 2;18(8):e0012388. doi: 10.1371/journal.pntd.0012388 (PMC11333008; doi:10.1371/journal.pntd.0012388)

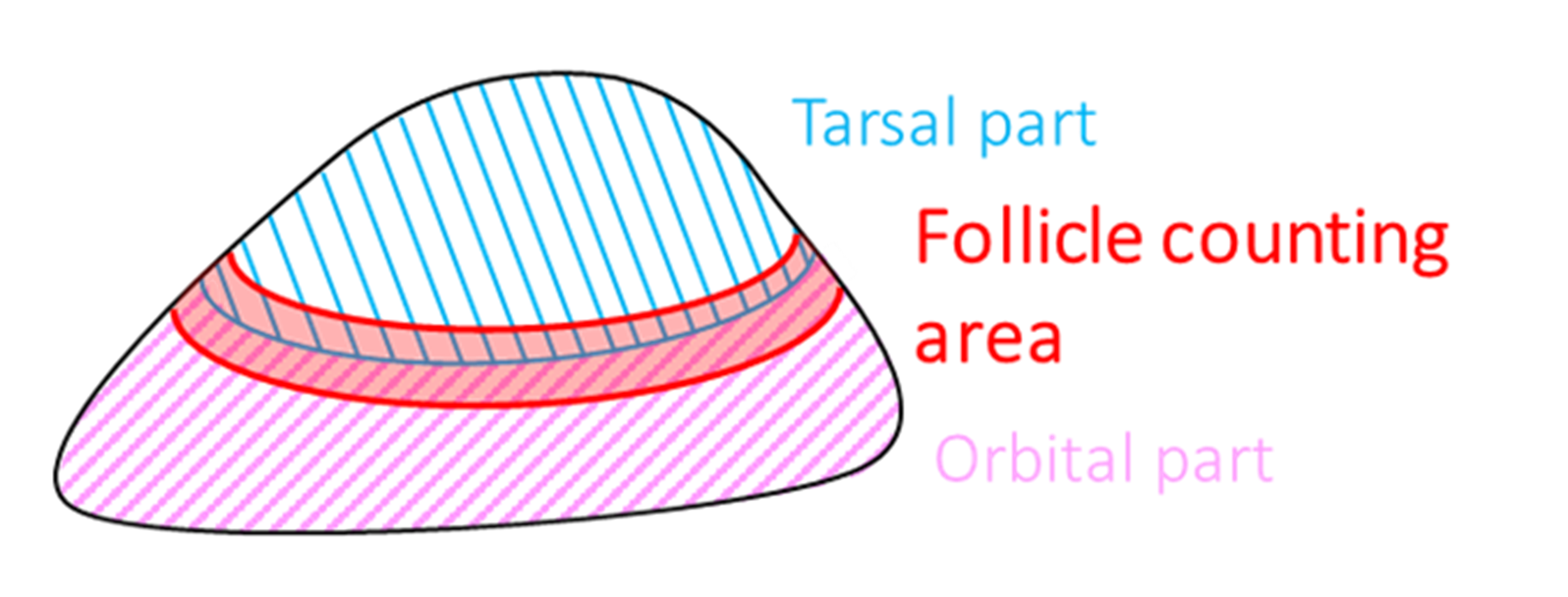

Supplement: S1 Fig — Follicle counting area is shown in red at the interface of the tarsal part (blue) and the orbital part (pink) of the palpebral conjunctiva. (TIF) [file pntd.0012388.s001.tif]

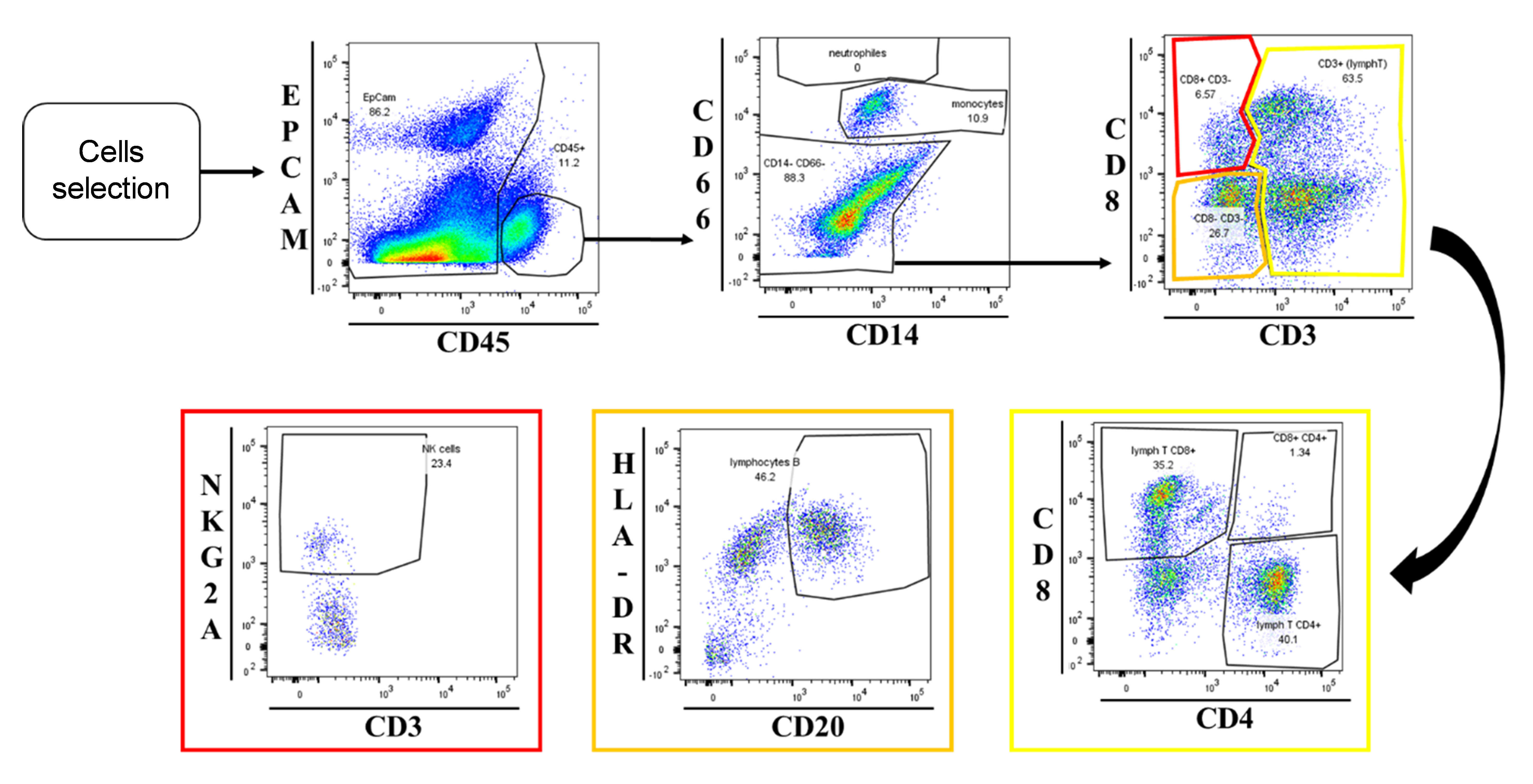

Supplement: S2 Fig — This strategy was used to assess the following ocular surface cell immune populations: leukocytes (CD45+), neutrophils (CD66+), monocytes (CD14+), T cells (CD3+), CD4 T cells (CD3+, then CD4+/CD8-), CD8 T cells (CD3+, then CD4-/CD8+), natural killer cells (CD3-/CD8+, then NKG2A+), B cells (CD20+ and HLA-DR+). This strategy is applied to conjunctival imprints’ cells’ flow cytometry data. (TIF) [file pntd.0012388.s002.tif]

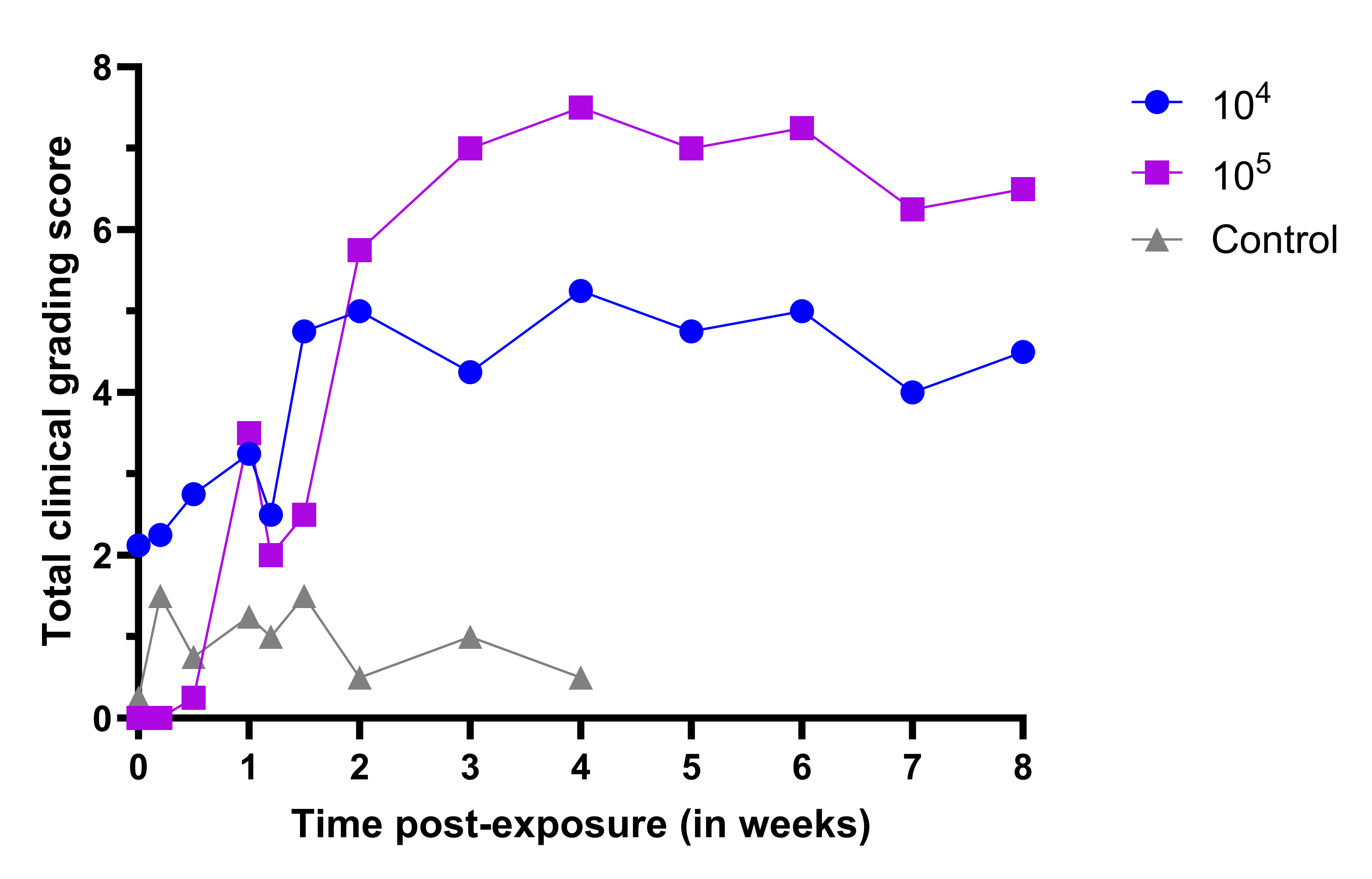

Supplement: S3 Fig — Each line represent the mean of 4 clinical scores (one score per eyes and two animals per subgroup) of animals respectively exposed to 104 IFU of Ct (blue line), to 105 IFU of Ct (purple line), and only to the SPG buffer (grey line). (TIF) [file pntd.0012388.s003.tif]

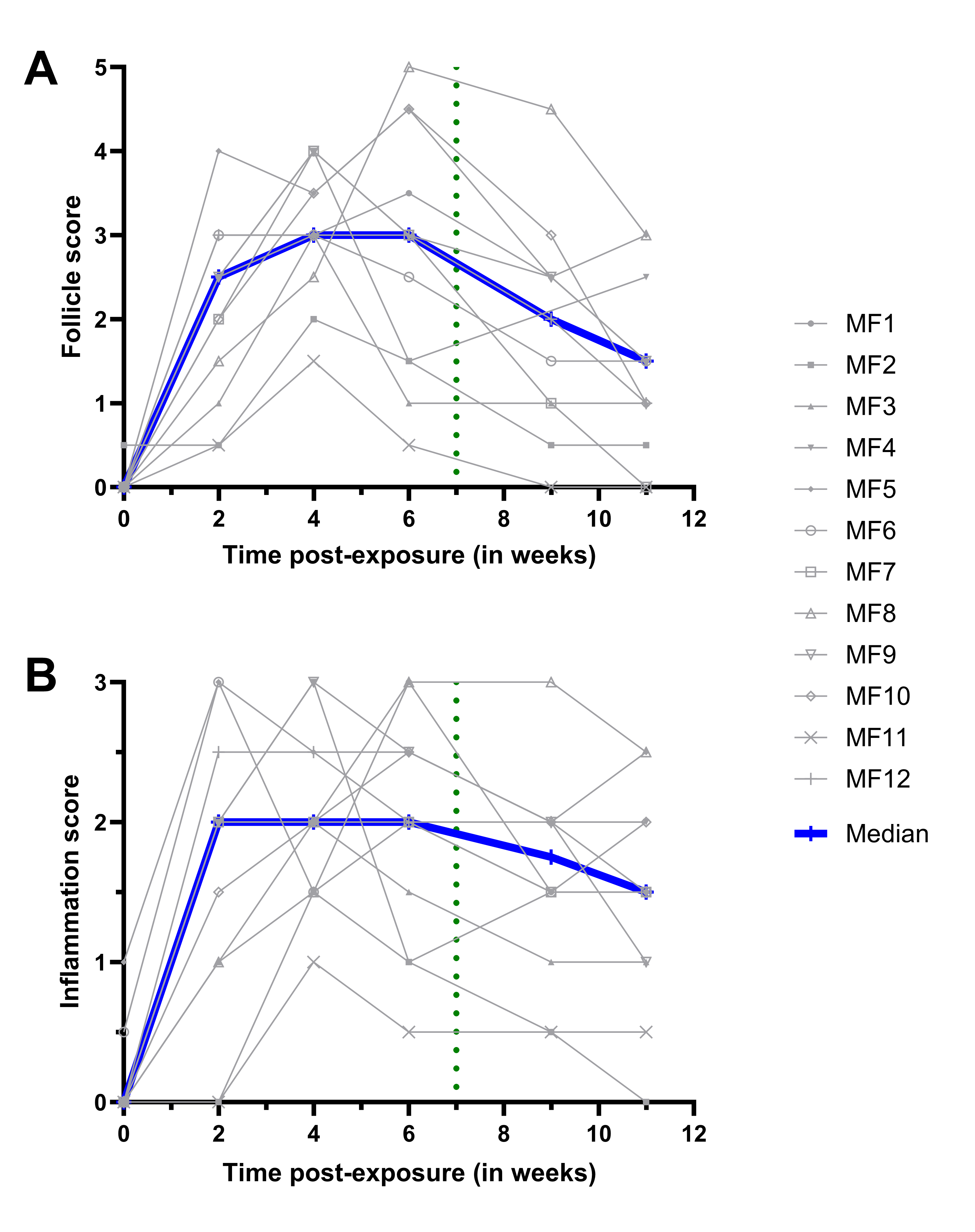

Supplement: S4 Fig — Conjunctival clinical scoring results for follicule score (A) and inflammation (B) in Group 2 (TIF) [file pntd.0012388.s004.tif]

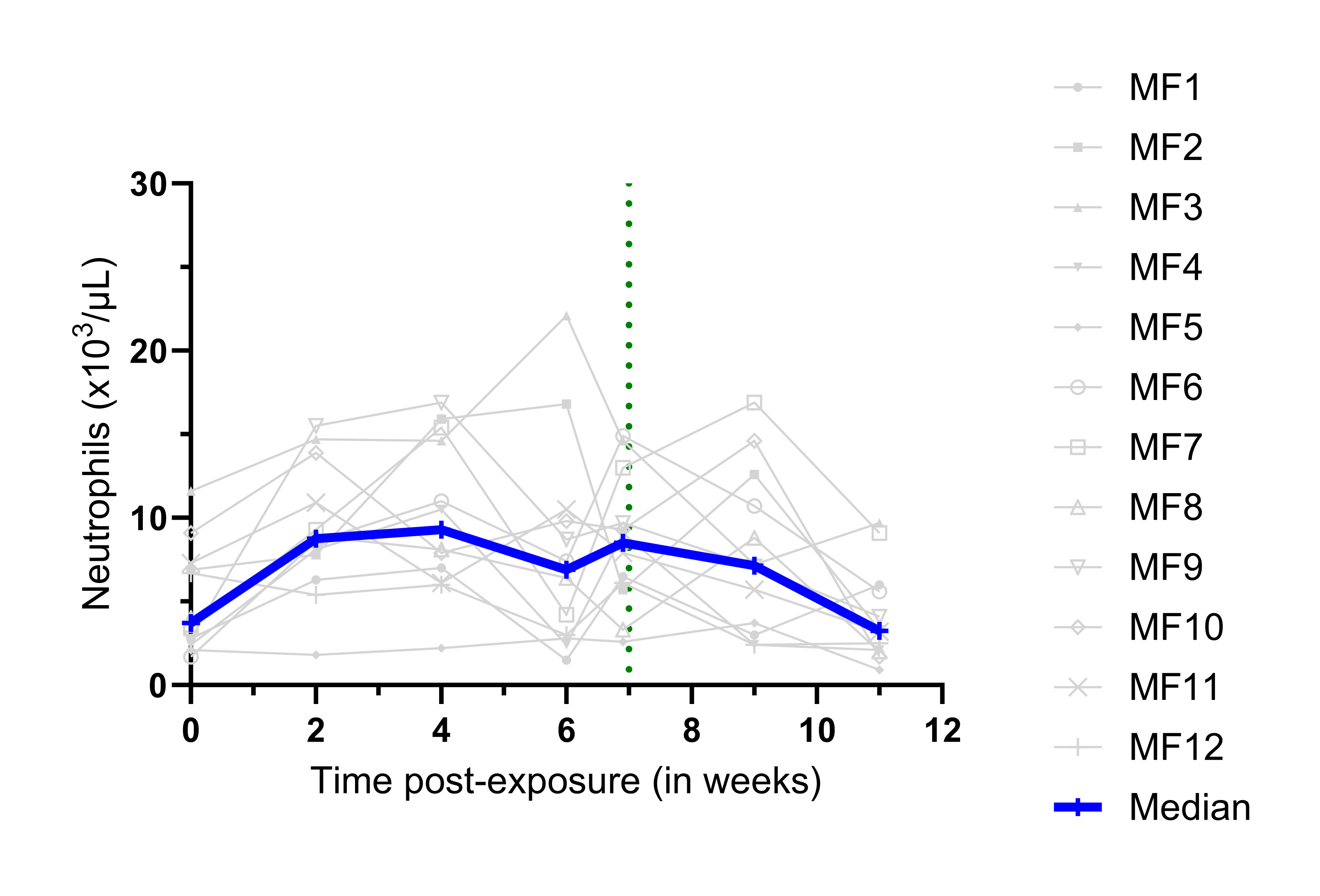

Supplement: S5 Fig — (performed at each time-point as part of a complete blood count. (TIF) [file pntd.0012388.s005.tif]

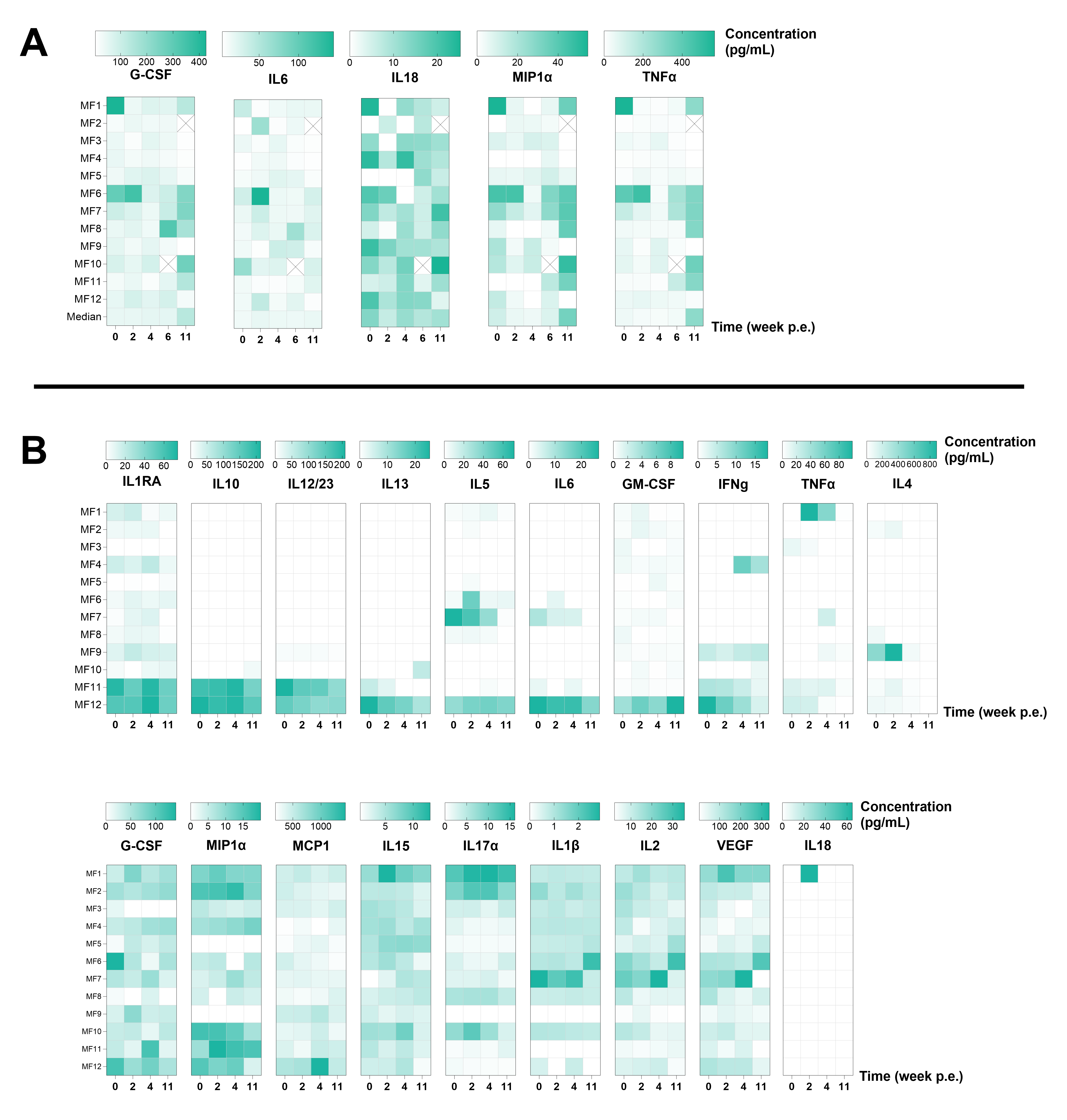

Supplement: S6 Fig — Cytokines quantification without statistically significant changes quantified on tears (A) and serum (B). The crossed boxes represent missing data. (TIF) [file pntd.0012388.s006.tif]
